# Development of a Laser Scanning Machining System Supporting On-the-Fly Machining and Laser Power Follow-Up Adjustment

**DOI:** 10.3390/ma15165479

**Published:** 2022-08-09

**Authors:** Yisheng Yin, Chengrui Zhang, Tieshuang Zhu, Liangcheng Qu, Geng Chen

**Affiliations:** 1 Key Laboratory of High Efficiency and Clean Mechanical Manufacture (Ministry of Education), School of Mechanical Engineering, Shandong University, Jinan 250061, China; 2 National Demonstration Center for Experimental Mechanical Engineering Education, Shandong University, Jinan 250061, China

**Keywords:** laser scanning machining, galvanometer scanner, on-the-fly machining, laser power follow-up adjustment

## Abstract

In this study, a laser scanning machining system supporting on-the-fly machining and laser power follow-up adjustment was developed to address the increasing demands for high-speed, wide-area, and high-quality laser scanning machining. The developed laser scanning machining system is based on the two-master and multi-slave architecture with synchronization mechanism, and realizes the integrated and synchronous collaborative control of the motion stage or robot, the galvanometer scanner, and the laser over standard industrial ethernet networks. The galvanometer scanner can be connected to the industrial ethernet topology as a node, via the self-developed galvanometer scanner control gateway module, and a “one-transmission and multiple-conversion” approach is proposed to ensure real-time ability and synchronization. The proposal of a laser power follow-up adjustment approach could realize real-time synchronous modulation of the laser power, along with the motion of the galvanometer scanner, which is conducive to ensuring the machining quality. In addition, machining software was developed to realize timesaving and high-quality laser scanning machining. The feasibility and practicability of this laser scanning machining system were verified using specific cases. Results showed that the proposed system overcame the limitation of working field size and isolation between the galvanometer scanner controller with the stage motion controller, and achieved high-speed and efficient laser scanning machining for both large-area consecutively and discontinuously arrayed patterns. Moreover, the integration of laser power follow-up adjustment into the system was conducive to ensuring welding quality and inhibiting welding defects. The proposed system paves the way for high-speed, wide-area, and high-quality laser scanning machining and provides technical convenience and cost advantages for customized laser-processing applications, exhibiting great research value and application potential in the field of material processing engineering.

## 1. Introduction

With the development of laser machining technology, laser machining technology has been widely applied to numerous industrial applications, such as welding [1], cladding [2], drilling [3], cutting [4], and micro-structure manufacturing [5]. For mass or small-batch customized production, a laser machining technique that meets the requirements of high-speed, wide-area, and high-quality must be developed, and the advent of laser scanning machining technology greatly assists in this endeavor [6]. As a new manufacturing technology in the field of materials processing engineering, laser scanning machining technology has attracted much attention. It can quickly and accurately change the laser focus position using a galvanometer scanner, so that the spots with different energy densities are focused on different positions of the workpiece to complete the processing. Laser scanning machining technology has many advantages over traditional laser processing methods, including high dynamic performance and processing speed [7], great potential to improve tolerances of joint gaps [8], and high machining quality [9]. 

However, for laser scanning machining systems, which usually consist of a laser and a galvanometer scanner, wide-area machining is challenging, due to the working field size of the galvanometer scanner, which is limited by the focal length of the f-theta objective and the scanning stroke. Even if the focal length is increased, the working field size would then widen, but precise fabrication would not be possible due to the reduced resolution of the scanning field [10]. On the other hand, in some applications of laser scanning machining, the laser spot could be periodically oscillated by the scanning motion of the galvanometer scanner to improve the machining quality. However, the variation of local effective machining speed could lead to many defects, due to the variation of linear energy distribution [11,12]. Thus, the major challenge of laser scanning machining is combining various methods to focus the laser beam on the workpiece to break through the limitation of the working field size, and to realize the laser power follow-up adjustment through synchronously and precisely modulating the laser output power, along with the motion of the galvanometer scanner [13]. On the basis of two traditional methods, namely, moving the workpiece under a fixed laser and moving the laser beam over a fixed workpiece, the conventional step-scan method was developed for breaking through the limitation of the working field size [14]. In the step-scan method, the laser scanning machining system introduces the motion stage or robot composed of multi-axis mechanisms, the stage (or the robot) moves to a specified position in turn, and the galvanometer scanner operates when the stage (or the robot) stops. The repetitive start–stop motion of the stage (or the robot) and scanner can lead to stitching errors, resulting in poor surface quality at the boundaries of the scanning areas. In addition, the fabrication time is significantly increased during high-speed and wide-area machining [15]. A great amount of research effort has been devoted to compensating or eliminating the stitching errors and improving the efficiency. For example, Ai et al. presented a cross-scale laser lithographic system, using the chart-based compensation method [16], and Delgado et al. proposed a calibration method to reduce lens distortion, based on a visual aid system [17]. However, none of these are ways to avoid stitching errors and improve efficiency in principle. In order to break through the limitation of the working field size, eliminate non-production time and avoid stitching errors, a synchronous motion control architecture, in which the scanner and stage can move simultaneously, is necessary [15]. An on-the-fly method that involves moving the workpiece and the laser beam simultaneously was developed recently. Kim et al. proposed a method of synchronizing the motion of the scanner and the linear stage, and verified the feasibility of the method by laser flying marking experiments [18]. Yoon et al. proposed a one-axis on-the-fly system for wide-area laser machining [19]. Martinez et al. applied the on-the-fly method to remote laser hardening machining [20]. Stoesslein et al. used the on-the-fly pulsed laser machining method to reduce the surface roughness of a workpiece [21]. Buser et al. proposed a scanning path strategy for improving fabrication efficiency [22]. Jiang et al. presented and compared the maximum flying marking velocity using different scanning path algorithms [23]. Kim et al. also proved that the on-the-fly method can improve quality and throughput and simplify optic requirements [10]. 

Notably, in most current commercialized laser scanner machining systems supporting the on-the-fly method, the galvanometer scanner controller is isolated from the motion controller, which is typically used to control the motion of the linear stage or robot. The reason for this situation is that the motion controller and the galvanometer scanner’s controller are designed to operate on different principles. For example, motion control comes from the background of combining long travel motorized platforms with rotary spindle control at different speeds, while the galvanometer scanner controller is designed for high speed and high frequency motion control and laser control over a relatively short range of travel. Hence, the methodology of programming each controller varies in terms of language, parameters, capabilities, and historical background. It also results in the galvanometer and motion stage being controlled by different servo systems, making it difficult to synchronize the fabrication process. In addition, the conventional galvanometer scanner controller, based on an embedded system, has limited flexibility and ease of use in combining the scanning motion with the precise modulation of laser power, and it is difficult to achieve laser power follow-up adjustment. Very recently, a motion stage and galvanometer scanner were reported to achieve integrated and synchronized control, based on standard industrial ethernet networks [14], but the connection between the stage and scanner is still based on two control boards, and the isolation between them still exists. Moreover, the whole system is not based on open architecture, and, thus, it is unsuitable for customized laser-processing applications. 

In this study, our self-developed laser scanning machining system can overcome these difficulties and challenges, which include the limitation of the working field size, the laser power follow-up adjustment, and the isolation between the galvanometer scanner controller with the stage motion controller, to meet the requirements of high-speed, wide-area, and high-quality laser scanning machining. In our self-developed system, the control commands of the motion stage, the galvanometer scanners and laser are both generated by the PC-based controller, and sent through the standard industrial ethernet network in each communication period to support on-the-fly machining and laser power follow-up adjustment. The system is based on the two-master and multi-slave control structure with synchronization mechanism. The galvanometer scanner drive units connect to the industrial ethernet network via the self-developed galvanometer scanner control gateway module and the “one-transmission and multiple-conversion” approach to ensure real-time ability and synchronization. Other contributions of this study include the proposal of a laser power follow-up adjustment approach, which synchronously and precisely modulates the laser output power along with the motion of the galvanometer scanner to improve the processing quality, and the development of machining software to realize timesaving and high-quality laser scanning machining. In addition, the open architecture-based system also provides convenience for customized laser-processing applications. The feasibility and practicability of this laser scanning machining system were verified through specific cases. These cases reflected the great potential of the proposed laser scanning machining system for high-quality and high-efficiency laser material processing.

## 2. Architecture and Method

### 2.1. Two-Master and Multi-Slave Architecture with Synchronization Mechanism

**Figure 1:**
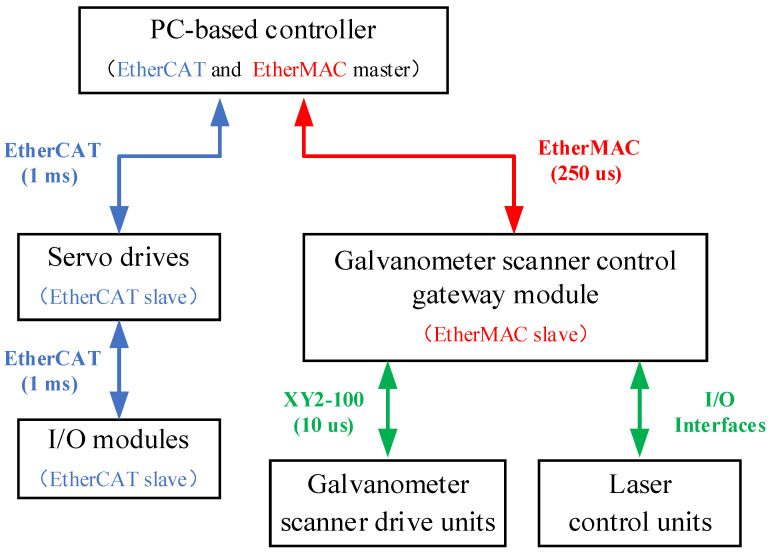
Two-master and multi-slave architecture.

Considering the difference of the on-the-fly method compared with the existing the step-scan method, and that the motion stage and the galvanometer scanner have quite different application conditions, the architecture of the laser scanning machining system is based on the two-master and multi-slave control structure and is shown in Figure 1. The system is composed of a PC-based controller (self-developed), a galvanometer scanner control gateway (self-developed), servo drives, and digital and analog input and output (I/O). Among them, the PC-based controller acts as the master nodes on the EtherCAT industrial ethernet network (Beckhoff Automation GmbH &Co. KG, Verl, Germany) and the EtherMAC industrial ethernet network (Shandong University, Jinan, China) [24]. In addition, the gateway module, the servo drives, and I/O modules act as the slave nodes. The EtherMAC industrial ethernet network, with independent intellectual property rights proposed by our group, is a real-time ethernet communication solution based on ethernet, and this solution employs the master/slave structure. The slave node is based on a field programmable gate array (FPGA) and manages the communication cycle. Moreover, a relatively distributed clock mechanism is also adopted to realize accurate synchronization between slave nodes [25].

In the laser scanning machining system, on the one hand, the PC-based controller communicates with the corresponding servo drives and the I/O modules through the EtherCAT, where the communication period is set to 1 ms, and the servo drivers and the I/O modules act as the slave nodes on the EtherCAT network. On the other hand, the galvanometer scanner drive units and the laser control units connect to the EtherMAC network via the galvanometer scanner control gateway module, which was independently developed and based on Altera EP4CE6E22C8 FPGA (Altera Corporation, San Jose, CA, USA), where the communication period is set to 250 us, and the gateway module acts as a slave EtherMAC node. 

As shown in Figure 1, as the master node, the PC-based controller in this system calculates the control commands of each slave node and sends the network frame to the corresponding slave node through the industrial ethernet network. In each communication period, the master node (the PC-based controller) exchanges data with the slave nodes, ensuring continuous motion stage (or robot) movement, real-time signal transfer between the motion stage (or the robot) and the galvanometer scanner, and laser control. The gateway module extracts the relevant commands of the galvanometer scanner and laser from the EtherMAC network frame. Among them, the scanner commands are converted to a serial bus protocol (XY2-100) that the scanner can interpret, namely, protocol conversion, and the laser control commands are used to control the laser. In addition, according to the received control commands from the EtherCAT network frame, the servo drives are used to drive the motion stage or the robot and the I/O modules are used for switching interfaces and other peripheral equipment in laser scanning machining. 

The operating system of the PC-based controller is Windows 10 (Shandong University has obtained the genuine license for Windows 10 operating system), which is extended by the Kithara real-time suite (Shandong University has obtained the genuine license for the Kithara real-time suite). The Kithara real-time suite (KRTS) can provide excellent real-time performance for the Windows operating system and support the EtherCAT and the EtherMAC real-time industrial ethernet communication protocol. The Kithara EtherCAT Master module is part of the Kithara real-time suite and acts as a function library to implement complex automation tasks [26]. Through the KRTS, the operating system of the PC-based controller can be divided into two sub-systems: the non-real-time operating system (non-RTOS), which can perform the tasks with no real-time requirements, and the KRTS-Kernel, which is a real-time system with excellent real-time performance. The HMI task of this system can be implemented in the non-RTOS. The real-time collaborative interpolation of the motion stage (or the robot) and the galvanometer scanner (and the laser power) is conducted in the KRTS-kernels, specifically, in the EtherCAT kernel and EtherMAC kernel. Considering the difference between the communication period and clock of EtherCAT industrial ethernet network and EtherMAC industrial ethernet network, while using shared memory and ring buffer to complete communication between EtherCAT kernel and EtherMAC kernel, the mechanism of kernel counting and event synchronization is adopted to overcome the error and the jitter caused by non-synchronization of real-time industrial ethernet communication clocks, and to ensure the real-time and synchronization of the system. In addition, the open-architecture-based system also provides convenience for customized laser-processing applications.

### 2.2. One-Transmission and Multiple-Conversion Approach

**Figure 2:**
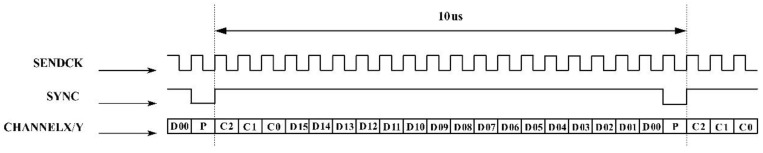
Transmission sequence of XY2-100 protocol.

The control interface of the galvanometer scanner is the key to the integration of the galvanometer scanner and stage (or robot) motions. XY2-100 is the communication protocol of the digital galvanometer scanner. The XY2-100 protocol includes four differential signals: SENDCK (clock signal), SYNC (synchronization signal), CHANNELX (X-channel data), and CHANNELY (Y-channel data) [27]. The transmission of these four differential signals is based on a synchronous serial transmission pattern, and the transmission sequence is shown in Figure 2. Among the signals, the frequency of the SENDCK signal is 2 MHz, CHANNELX/Y is composed of 20 bits, and the control cycle of the scanner is 10 us. In addition, the STATUS signal is sent by the galvanometer scanner; the STATUS output is not synchronized with the SENDCK input. Thus, the major challenge of the integrated control is the limitation of the PC-based controller’s control cycle, which is unable to be realized up to 10 us.

**Figure 3:**
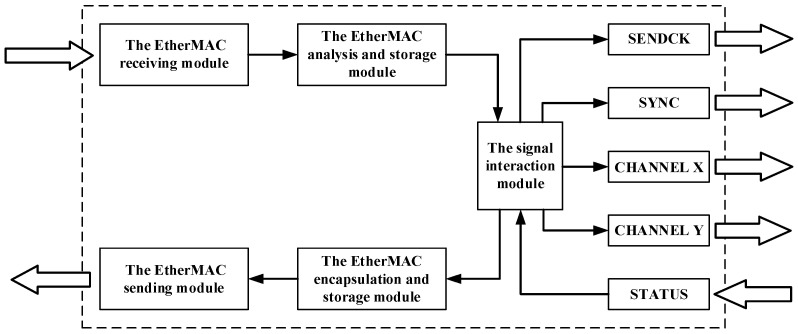
Design diagram of the gateway module.

To overcome this challenge, as mentioned in Section 2.1, the galvanometer scanner is connected to the EtherMAC network by the gateway module to achieve the integrated control by standard industrial ethernet networks. The communication period of the galvanometer scanner control gateway module, which acts as the slave node on the EtherMAC network and as the controller for the galvanometer scanner, is set to 250 us. The gateway module uses the FPGA as the main control chip for protocol conversion and utilizes the characteristics of FPGA parallel execution. The design diagram of the gateway module is shown in Figure 3. The EtherMAC receiving and sending modules complete the receiving and sending of real-time industrial ethernet network data frames according to the real-time synchronization mechanism [24]. The analysis and storage modules are responsible for analyzing the relevant commands of the galvanometer scanner in the network frame. The encapsulation and storage modules are responsible for encapsulating the returned galvanometer scanner status to the sending module. The signal interaction module is responsible for sending four control signals and receiving status signals, according to the XY2-100 protocol.

However, the communication period of the galvanometer scanner control gateway module is set to 250 us, and the communication period of the galvanometer scanner, which communicates with the gateway module via the XY2-100 serial buses, is 10 us, so the former is 25 times the latter. Therefore, the “one-transmission-multiple-conversion” approach is used to align the communication period. In each communication cycle of the gateway module, the PC-based controller calculates the galvanometer scanner control and laser commands of the corresponding multiples and encapsulate them in the same network frame. The gateway module receives a network frame once, and issues the instructions in multiple sequential conversions. In this manner, on the premise of ensuring real-time ability and synchronization, the galvanometer scanner can be connected to the industrial ethernet topology as a node and achieve real-time signal transfer with the motion stage (or the robot), the laser and the other devices via the industrial ethernet.

### 2.3. Laser Power Follow-Up Adjustment Approach

Combining the scanning motion of laser spot with the precise modulation of laser output power could change the laser energy deposition distribution on the processing surface [13]. Therefore, the real-time synchronous adjustment of the laser power and galvanometer scanner motion should be considered in laser scanning processing. To dynamically adjust the laser power with the scanning speed of the galvanometer scanner in real time and improve real-time ability and accuracy of the follow-up adjustment as much as possible, the self-developed gateway module integrates the laser control and galvanometer control interfaces in our self-developed system. The laser control logic and the power follow-up adjustment are completed by the PC-based controller, real-time mixed interpolation is carried out for the laser power and the galvanometer scanner motion in the interpolation cycle, and the interpolation results are encapsulated in the corresponding network frame. The gateway module is only responsible for analyzing and transmitting the instructions in the network frame sent by the PC-based controller. The laser power follow-up adjustment interface optimizes the adjustability of the laser scanning machining process parameters, improves real-time ability and synchronization of the laser power adjustment, further improves the system performance, and ensures processing quality.

## 3. Experiments and Discussion

### 3.1. Experimental Devices

**Figure 4:**
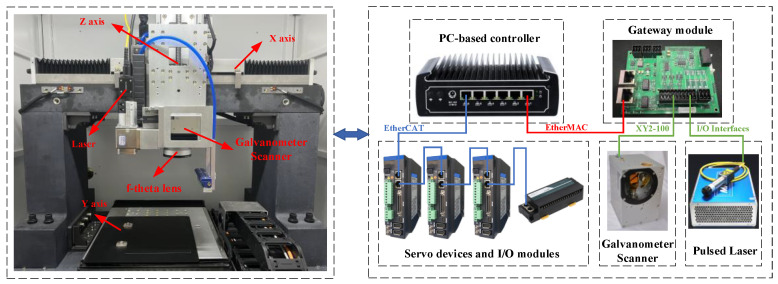
Scheme of the experimental setup based on scanner and stage.

As shown in Figure 4, an experimental setup was developed to undertake a wide-area and high-speed laser scanning machining experiment. A 2D scanner head with a scanning range of 100 mm × 100 mm, an aperture of 14 mm and a focal length of 330 mm was mounted on a three-axis motion platform. A pulsed laser source was used to generate a laser beam with a maximum power of 20 W and a central wavelength of 1064 nm. In the three-axis motion platform, the range of the direct drive linear motion stage as the X and Y axes was 400 mm × 400 mm, the maximum speed was 1000 mm·s^−1^, and the range of the leading screw module as the Z axis was 0~400 mm. In addition, the experimental setup also included a PC-based controller (self-developed), and a galvanometer scanner control gateway (self-developed).

**Figure 5:**
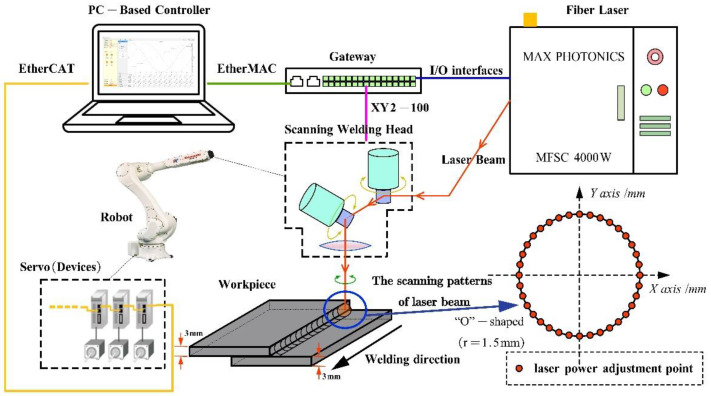
Scheme of the experimental setup based on scanner and robot.

Another experimental setup (Figure 5) included a Kawasaki six-axis industrial robot, a scanning welding head, a fiber laser in the continuous beam mode, a PC-based robot controller (self-developed), and a galvanometer scanner control gateway (self-developed). The scanning welding head, which was driven by the robot to move in the XY plane, consisted of a collimation unit with a focal length of 125 mm, two galvanometer scanner units, and a 500 mm focal length non-telecentric f-theta field lens. The wavelength of the fiber laser was 1070 nm, the maximum power was 4000 W, and, after collimation, deflection and focusing, the radius of the focused spot was about 0.8 mm.

In this study, a scanning machining software was developed based on the C++ language, and run in Visual Studio 2013 and QT 5.7. The main functions of the software include importing CAD patterns, decomposing the path and the velocity, and controlling the laser, the galvanometer scanner, and the three-axis motion stage (or the six-axis industrial robot). 

### 3.2. Cases and Discussion

#### 3.2.1. Case 1: Scanning Machining for Large-Area and Consecutive Pattern 

To verify the function of the laser scanning machining system developed in this study, motion decomposition and on-the-fly scanning machining were carried out on the experimental setup based on the scanner and the stage (Figure 4) for fabricating a large-area and consecutive circle plus pentagram pattern. Considering that the circle plus pentagram pattern is composed of line patterns, sharp-angle patterns and circular patterns, these patterns are more representative in laser machining applications. The machining performance of the system can be reflected by processing these patterns, and it is easy to judge the processing quality through the intersections and boundaries of the circle plus pentagram pattern. Therefore, the large-area and consecutive circle plus pentagram pattern was used as the processing object in this case. This process included the following steps. 

Step 1, Distortion correction work: A distortion correction method assisted by machine vision was used for the distortion correction of the galvanometer scanner, and the distortion error was compensated online with a linear interpolation algorithm to eliminate the influence of the distortion of the galvanometer scanner under static conditions and improve the fabrication quality [28].

**Figure 6:**
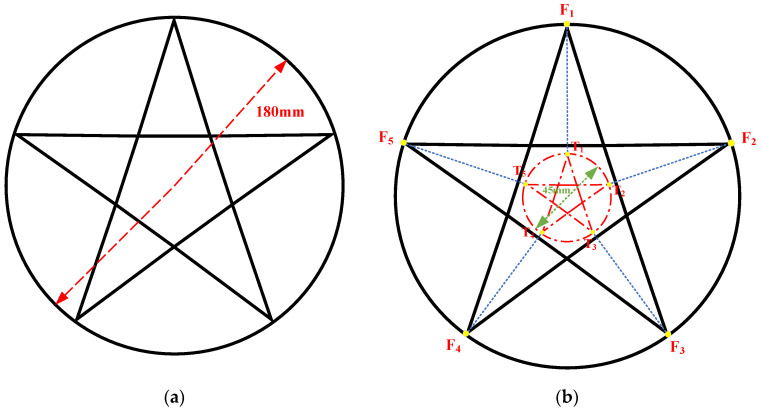
Schematic of the circle plus pentagram pattern and the motion decomposition result: (**a**) schematic of the fabricated trajectory; (**b**) the result of the motion decomposition.

Step 2, Path and velocity decomposition: As shown in Figure 6a, a circle plus pentagram pattern with a radius of 90 mm was selected as the fabricated trajectory. The trajectory was decomposed to the stage and scanner paths using the motion decomposition approach, and the control commands of the stage and the scanner were simultaneously transmitted by the PC-based controller. During motion decomposition, the stage allocated a path according to the principle of maximizing the working field of the galvanometer scanner because the galvanometer scanner enabled the delivery of the laser beam with near zero inertia, so that its velocity and acceleration were higher than those of the stage. The stage path could be determined by the working field size of the galvanometer scanner and the targeting CAD data. Once the stage path was determined, the scanner working path could be calculated from the vector relationship in Equation (1), where the fabrication movement vector ($\vec{p_{f}}$) was the summation of the stage movement vector ($\vec{p_{st}}$) and the scanner movement vector ($\vec{p_{sc}}$). To synchronize the motion of scanner and stage, the duration of each movement vector should be as shown in Equation (2), where the stage velocity ($V_{st}$) and the scanner velocity ($V_{sc}$) can be calculated by Equations (3) and (4), respectively. In this case, the motion decomposition result is shown in Figure 6b; the working field of the galvanometer scanner was set to 135 mm × 135 mm. The red circle plus pentagram pattern, with a radius of 22.5 mm, represented the path of the stage, and the lengths of lines $F_{1}T_{1}$, $F_{2}T_{2}$, $F_{3}T_{3}$, $F_{4}T_{4}$, and $F_{5}T_{5}$ satisfy Equation (5).

(1) $$\vec{p_{st}}+\vec{p_{sc}}=\vec{p_{f}}$$

(2) $$\frac{\left|\vec{p_{st}}\right|}{V_{st}}=\frac{\left|\vec{p_{sc}}\right|}{V_{sc}}=\frac{\left|\vec{p_{f}}\right|}{V_{f}}$$

(3) $$V_{st}=\frac{\left|\vec{p_{st}}\right|}{\left|\vec{P_{f}}\right|}\times V_{f}$$

(4) $$V_{sc}=\frac{\left|\vec{p_{sc}}\right|}{\left|\vec{P_{f}}\right|}\times V_{f}$$

(5) $$\left|F_{1}T_{1}\right|=\left|F_{2}T_{2}\right|=\left|F_{3}T_{3}\right|=\left|F_{4}T_{4}\right|=\left|F_{5}T_{5}\right|=67.5\;\mathrm{mm}$$

**Figure 7:**
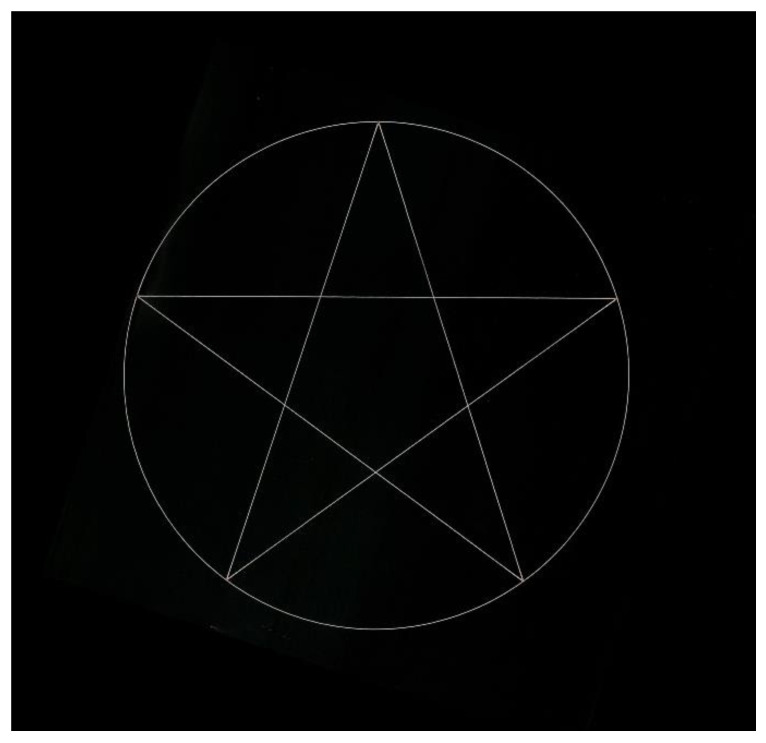
Result of laser scanning machining for large-area and consecutive pattern.

Step 3, Scanning machining: A laser with a power of 3 W was used to mark the large-area and consecutive circle plus pentagram patterns on the laser dimming paper. The marking speed was 1000 mm/s, and the velocity of the stage was calculated according to the manner described in Step 2. The process parameters of this case are summarized in Table 1. After in-situ machining, the designed patterns could be accurately marked, as shown in Figure 7. The machining results indicated that the processed patterns were complete and had no obvious flaws, and the position and size of the machined patterns were consistent with the design in the CAD software. Compared with the traditional machining method, based on stage motion, and fixed laser, the machining speed was greatly improved, due to the addition of the galvanometer scanner system. Moreover, the speed of the stage was only the same as that before the addition of the galvanometer scanner system. In this case, the marking speed was up to 1000 mm/s, while the stage speed was only 250 mm/s, and the marking speed was four times higher than that of the traditional machining method. Thus, this case reflected the great potential of the proposed laser scanning machining system for laser material processing with high speed and efficiency.

#### 3.2.2. Case 2: Comparison of Step-Scan Method with On-the-Fly Method 

**Figure 8:**
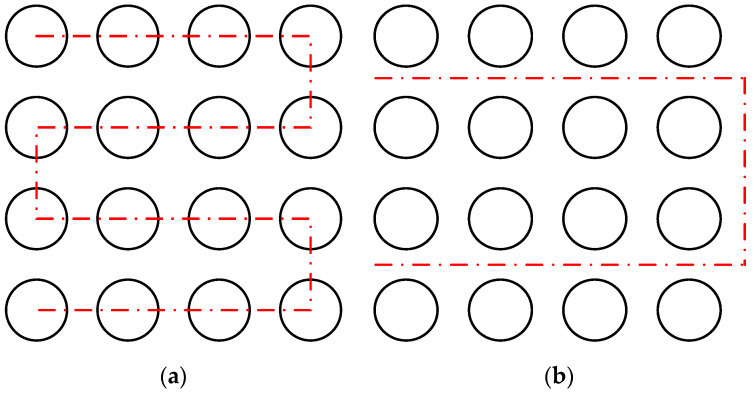
Schematic of the fabricated arrayed pattern and the different stage path generation manner: (**a**) schematic of the fabricated trajectory and stage path based on the step-scan method; (**b**) schematic of the fabricated trajectory and stage path based on the on-the-fly method.

When the fabricated pattern is discontinuous and arrayed, only the region that must be processed should be scanned to further reduce the workload. In this case, a large-area, discontinuous, and with an arrayed pattern was selected, as shown in Figure 8a. The meta trajectories were discontinuous with each other, and each meta trajectory (each circle) was within the scope of the galvanometer. However, the overall trajectory was much larger than the scope of the galvanometer. The fabrication times of the step-scan and on-the-fly methods were compared at the scan speed of 500 mm/s and the step speed of 50 mm/s of the experimental setup based on the scanner and the stage (Figure 4). 

In the step-scan method, the stage moved to every specified position in turn, and the galvanometer scanner operated within the working area after the stage stopped, as shown in Figure 8a. The red dotted line represents the stage path. In terms of on-line processing time, the galvanometer system did not eliminate the non-productive time caused by waiting at the position to be processed, resulting in a reduction of production efficiency during the mass processing of the workpiece. However, during on-the-fly processing, the stage and the scanner moved and worked together, and the galvanometer system compensated for the fabricated trajectory during the dynamic movement of the stage, maximizing the on-line time of the laser beam. The entire process included the following steps: 

Step 1: Distortion correction work. In both step-scan or on-the-fly methods, distortion correction must be performed on the galvanometer scanner through the same method described in Case 1.

Step 2: Stage path generation and scanner motion compensation. The stage path for laser fabrication is determined by the stage path generation strategy. In the stage path generation strategy of this case, the PC-based controller considered the arrangement of the meta trajectories and planned the optimal motion path of the stage based on the “central axis” principle. The “central axis” principle refers to grouping the meta trajectories according to the principle of axial symmetry, generating the central axis of each group of the meta trajectories and connecting them. The stage path generation strategy in this case is shown in Figure 8b, in which the red dotted line represents the generated stage path according to the “central axis” principle. Moreover, the main idea of the scanner motion compensation method was to obtain the displacement per interpolation cycle of the stage in the moving process and to compensate for this displacement in the interpolation trajectory of the galvanometer scanner, so that the fabricated trajectory would not be deformed due to the dynamic movement of the stage.

**Figure 9:**
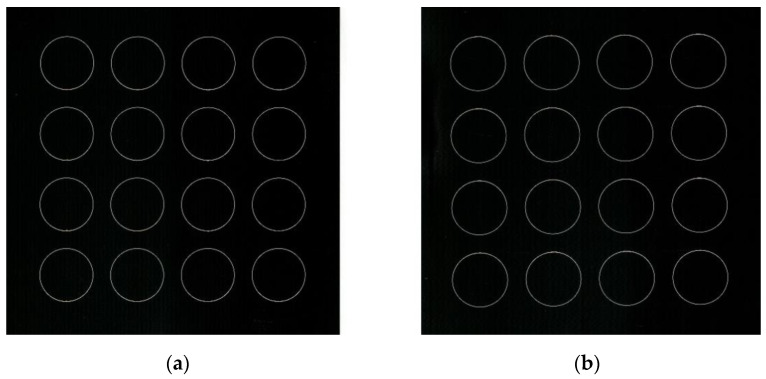
Result of laser scanning machining based on by different methods: (**a**) the step-scan method; (**b**) the on-the-fly method.

Step 3: Scanning machining. As shown in Figure 9a,b, a large-area discontinuous and arrayed pattern was fabricated on the laser dimming paper through both the step-scan and on-the-fly methods. The processing parameters of this case are summarized in Table 2. Compared with the step-scan method, after the stage path generation approach and the scanner motion compensation method are added, the stage must only move dynamically from the starting point to the end point, and the fabricated trajectory is realized during movement through the cooperation between the stage and the galvanometer scanner. The stage does not need to stop and wait for each processing to be completed, minimizing the non-productive time. The test showed that the processing time of the step-scan method was 24.2 s, while the processing time of the on-the-fly method was 10 s, indicating a reduction of 59%. Moreover, the speed of the stage was only 50 mm/s at this time. If the speed was increased, then the processing time would decrease. In addition, after adopting the on-the-fly method, the dynamic movement of the platform did not affect the processing quality. Thus, this case also reflected the great potential of the proposed laser scanning machining system for laser material processing with high speed and efficiency.

#### 3.2.3. Case 3: Laser Scanning Welding of 304 Stainless Steel Fillet Weld Lap Joint

Laser scanning welding technology emerged in recent years, and reportedly has the potential to improve the tolerances of joint gaps, microstructure homogeneity, and the weld quality [29,30]. In this case, the welding of 304 stainless steel (304SS) fillet weld lap joint under laser scanning tracks with an “O” shape scanning welding trajectory and laser power follow-up adjustment, described in Section 2.3, was achieved by the experimental setup based on a scanner and a robot (Figure 5). 

The welding experiments of fillet weld lap joint were conducted using 3-mm-thick 304SS plates measuring 200 × 100 mm^2^. The chemical composition is listed in Table 3. The 304SS plates were lap-welded according to the process parameters listed in Table 4. During welding, the weld surface was protected by pure argon, and the gas flow was 15 L/min. Before the experiment, the surfaces of the plates were washed with acetone, dried, polished to scrape off the oxide film, and washed with absolute ethanol. After welding, the weldment was cut, polished, and etched, and the relevant weld penetration characteristics were observed and measured under the VMA video measuring machine. 

Laser scanning welding can satisfy the welding requirements of fillet weld lap joint by adjusting the scanning amplitude to improve the adaptability of the welding gap and the welding quality [31]. However, “O”-shaped scanning welding leads to uneven laser energy distribution in the direction perpendicular to the weld, and a large amount of energy is concentrated on the edge of the weld, resulting in a large amount of base metal melting. Meanwhile, the energy at the center of the weld is low, and a continuous molten pool cannot be formed in a short time, resulting in poor penetration quality of the fillet weld lap joint and pore and undercut defects in weld section. Thus, the welding quality of the fillet weld lap joint cannot be ensured. 

**Figure 10:**
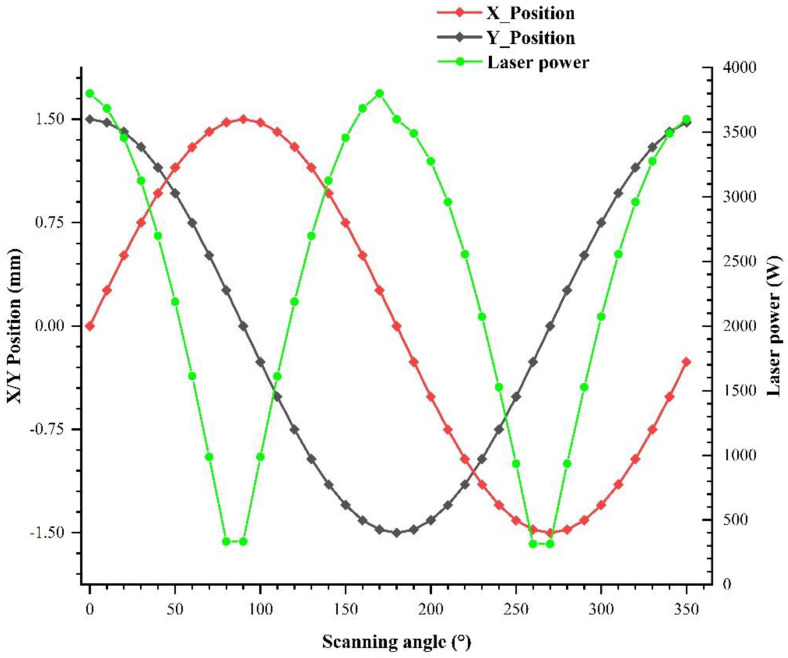
Laser power follow-up adjustment during the “O”-shaped scanning welding.

In this case, according to the principle that the total laser energy remained the same as when the laser power was not adjusted, and the linear energy density distribution was uniform in the direction perpendicular to the weld, the “O”-shaped scanning path was divided into 36 sections, as shown in Figure 5. The laser energy was redistributed, and the laser power of each section was adjusted, based on the analysis of the total laser energy, defocusing distance of lower plate and upper plate, and the linear energy density of “O”-shaped scanning welding along the direction perpendicular to the welding direction to control the penetration quality of the lap joint and to inhibit the defects. The constant laser power with no laser power follow-up adjustment, and the base laser power of lower plate and upper plate with laser power follow-up adjustment are listed in Table 3. Among them, considering the difference between the defocusing distance and the spot area of lower plate and upper plate, the base laser power of the lower plate was set to be different from that of the upper plate. The laser power follow-up adjustment during the “O”-shaped scanning welding is shown in Figure 10. 

**Figure 11:**
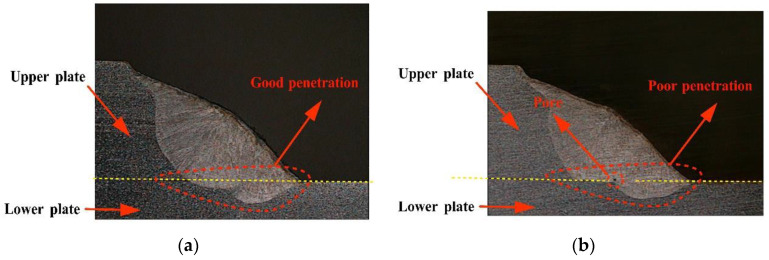
Result of the “O”-shaped scanning welding of 304 stainless steel fillet weld lap joint: (**a**) using the laser power follow-up adjustment; (**b**) no laser power follow-up adjustment.

In the PC-based controller, real-time mixed interpolation was carried out for the laser power and galvanometer scanner motion, ensuring real-time ability and synchronization of the laser power adjustment and galvanometer scanner motion. As shown in Figure 11a, the energy of the processing zone was controlled by the laser power follow-up adjustment, and the fillet weld lap joint was combined evenly. The penetration quality met the requirements, compared with the no laser power follow-up adjustment (Figure 11b), and the welding pool had good stability and an inhibitory effect on the pore defect. Thus, this case confirmed that the real-time synchronous adjustment of the laser power and the galvanometer scanner motion was conducive to ensuring the welding quality of the fillet weld lap joint and inhibiting welding defects.

## 4. Conclusions

In this study, a laser scanning machining system supporting on-the-fly machining and laser power follow-up adjustment was developed. The developed system overcame the major challenges of the current laser scanning machining system, including the limitation of the working field size, the laser power follow-up adjustment and the isolation between the galvanometer scanner controller with the stage motion controller. In cooperation with the developed machining software, convenient and timesaving laser scanning machining could be realized while meeting the requirements of high-speed, wide-area, and high-quality laser scanning machining. The following main conclusions can be derived:

1. The laser scanning machining system is based on the two-master and multi-slave architecture with synchronization mechanism. By means of the self-developed galvanometer scanner control gateway module and the “one-transmission and multiple-conversion” approach, the system overcomes the difficulties of the integrated and synchronous collaborative control which involves the motion stage or robot, the galvanometer scanner and laser, ensures real-time ability and synchronization of the system, as well as makes a breakthrough in combining the scanning motion with the precise modulation of laser power. It also provides technical convenience and cost advantages for customized laser-processing applications, due to being based on standard industrial ethernet and open architecture, which contribute to the system’s research value and application potential.
2. The results of the cases showed that the proposed system could achieve laser on-the-fly machining with high speed and efficiency for large-area and consecutive or discontinuous and arrayed patterns. Compared with the traditional machining method, based on the stage motion with the fixed laser and the step-scan method, in the cases, the machining speed of the on-the-fly machining was increased by 3 times and the processing time was reduced by 59%.
3. The laser power follow-up adjustment approach proposed in this study can combine the scanning motion of laser spot with the precise modulation of laser power. The results of the case showed that real-time synchronous adjustment of the galvanometer scanner motion and the laser power was conducive to ensuring welding quality and inhibiting welding defects attributed to the variation of linear energy distribution, which is crucial for high-quality laser scanning machining.
4. The feasibility and practicability of this laser scanning machining system for highspeed, wide-area and high-quality laser scanning machining were verified through the cases of laser flying marking and laser scanning welding. The system should expand with more process functions in the future and be validated in other laser scanning processing applications, such as laser scanning cutting, laser additive manufacturing, laser micro-structure manufacturing, etc. In addition, more new laser scanning machining processes should be further researched and developed, based on this system, in the field of materials processing engineering.

## Figures and Tables

**Table 1:** Process parameters of laser scanning machining for large-area and consecutive patterns.

Laser Power	Marking Speed	Stage Speed	Marking Acceleration	Stage Acceleration
3 W	1000 mm/s	250 mm/s	10,000 mm/s^2^	2500 mm/s^2^

**Table 2:** Process parameters of laser scanning machining for discontinuous and arrayed pattern.

Laser Power	Marking Speed	Stage Speed	Marking Acceleration	Stage Acceleration
3 W	500 mm/s	50 mm/s	5000 mm/s^2^	500 mm/s^2^

**Table 3:** Chemical composition of the 304SS plate.

C	Si	Mn	P	S	Cr	Ni	Cu	Fe
0.027	0.56	1.55	0.031	0.001	18.0	8.0	0.1	Bal.

**Table 4:** Welding parameters of the “O”-shaped scanning welding.

Welding Parameters	Value
Welding speed (mm/s)	8
Constant laser power with no laser power follow-up adjustment (W) Base laser power of lower plate with laser power follow-up adjustment (W) Base laser power of upper plate with laser power follow-up adjustment (W)	2300 3800 3600
Scanning frequency (Hz)	30
Scanning amplitude (mm)	3
The gas flow of the nozzles (L/min)	15
Defocusing distance of lower plate (mm) Defocusing distance of upper plate (mm)	+3 0
